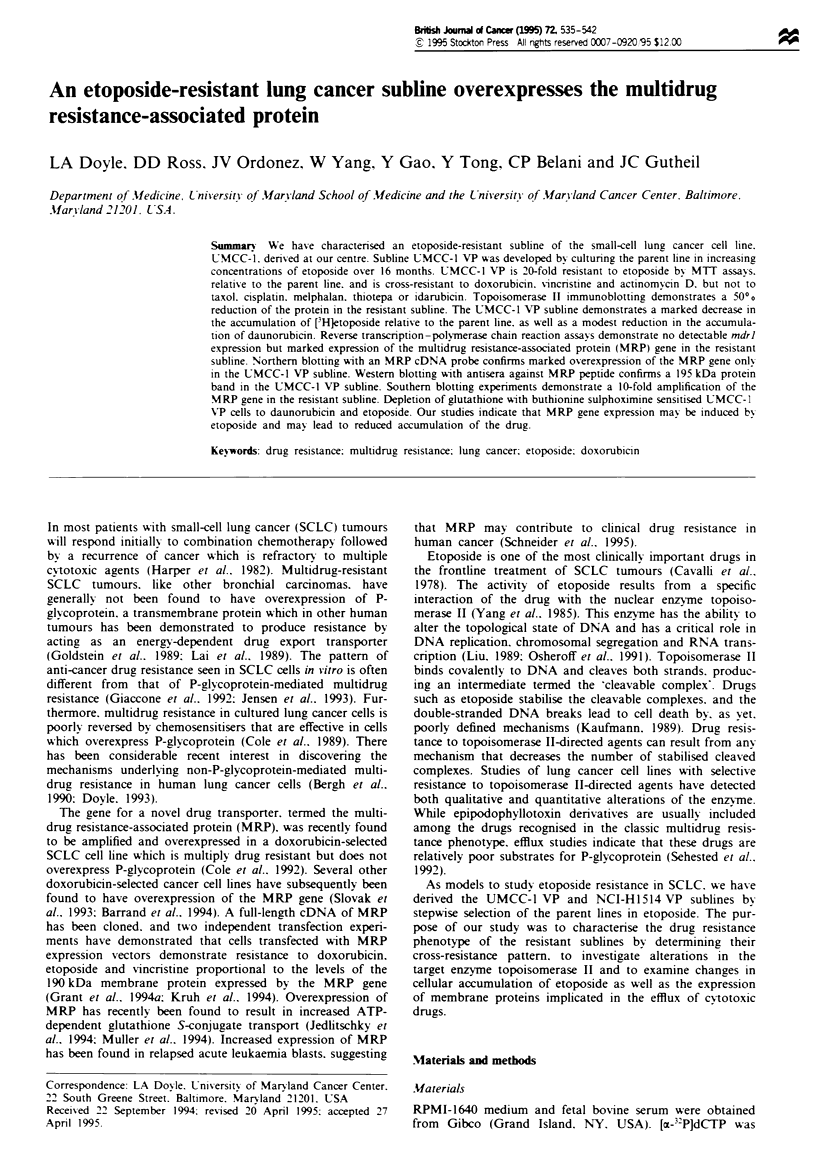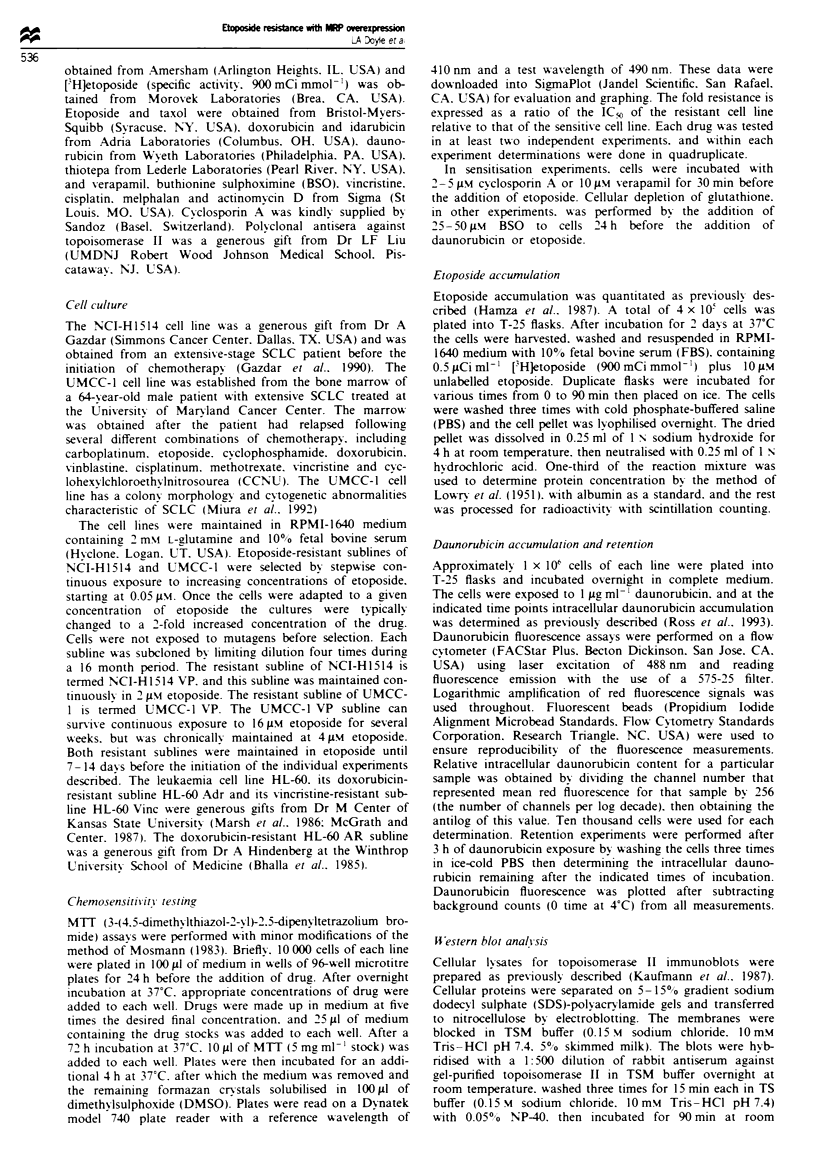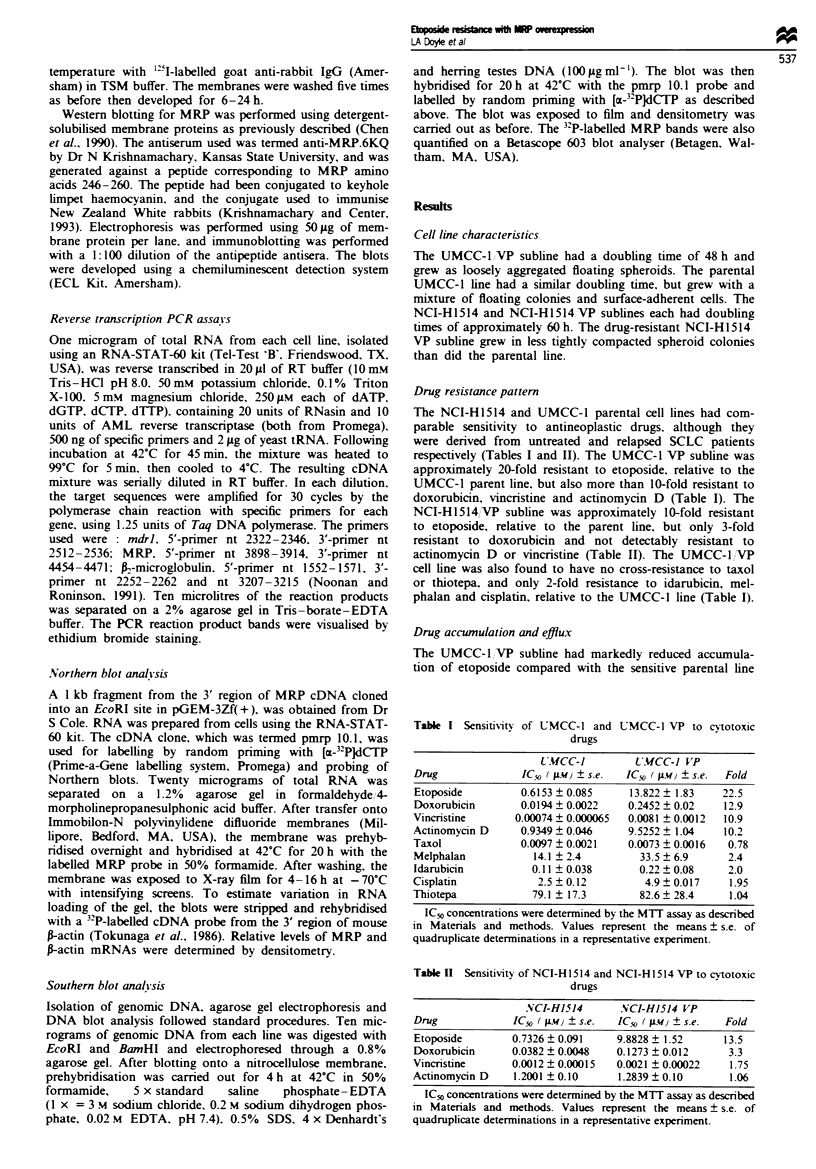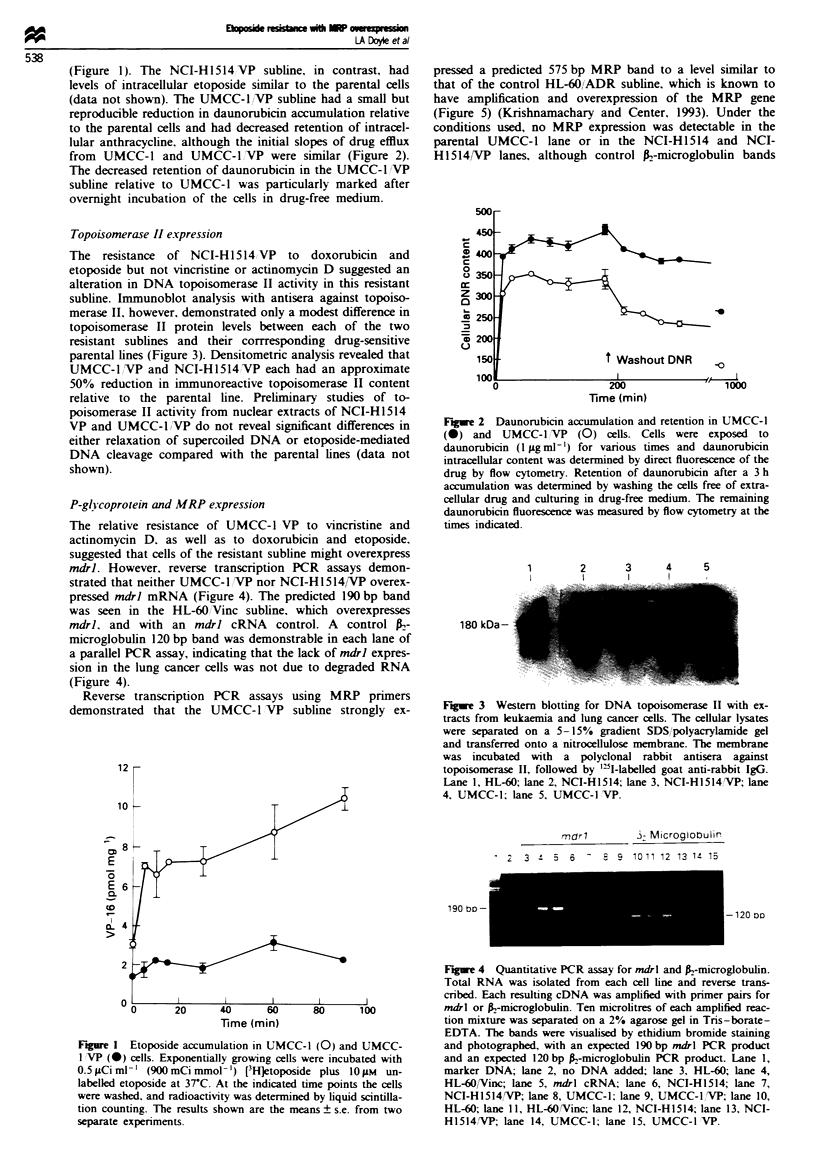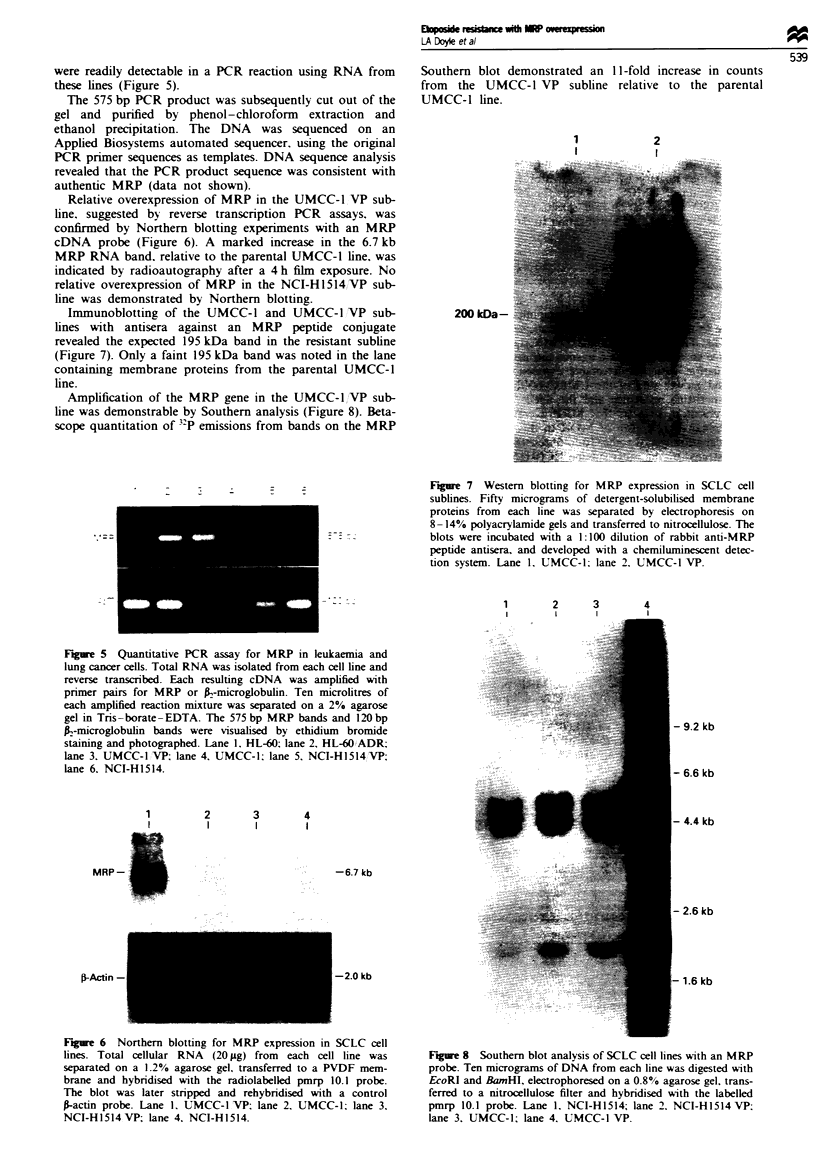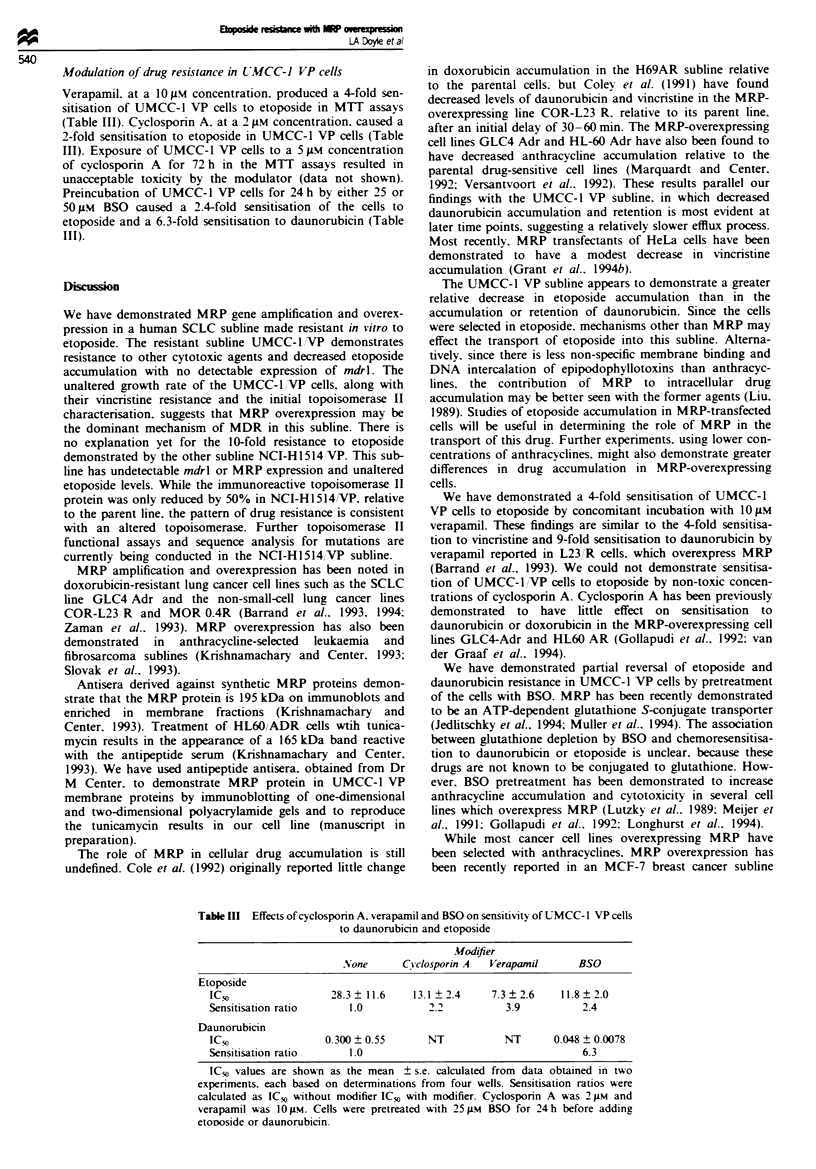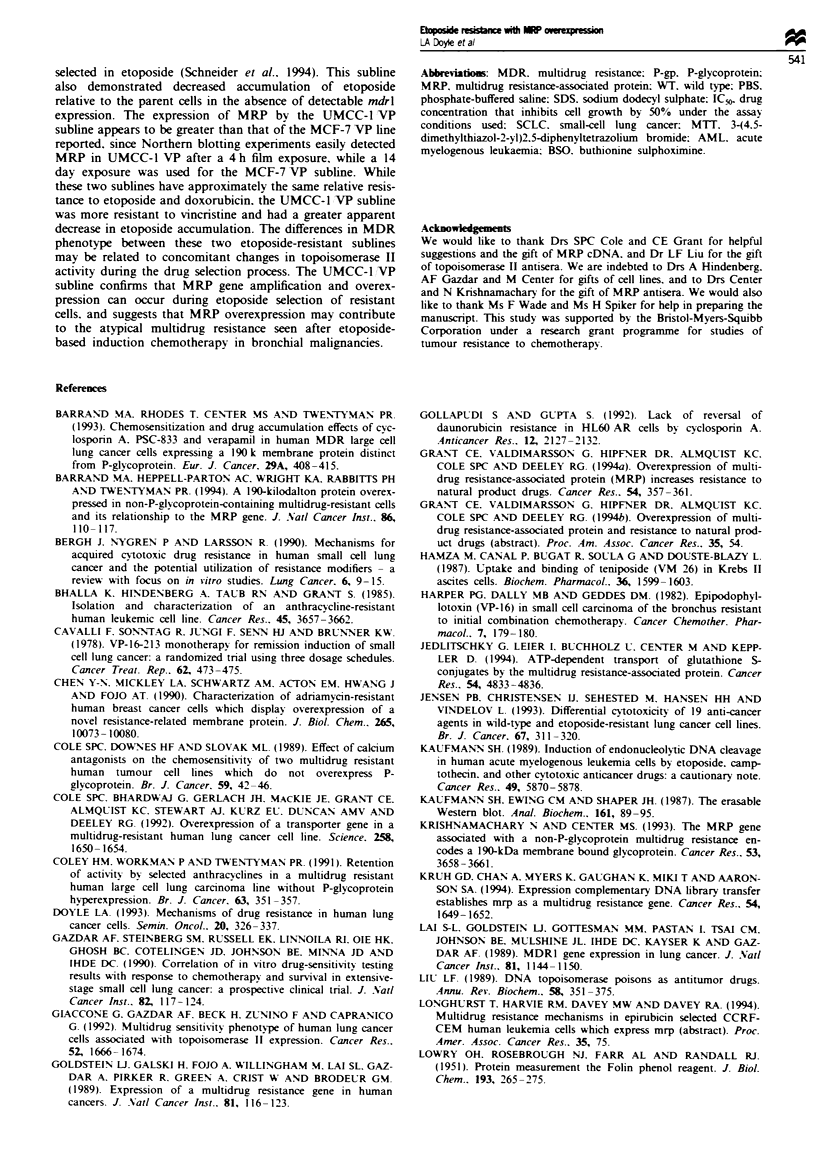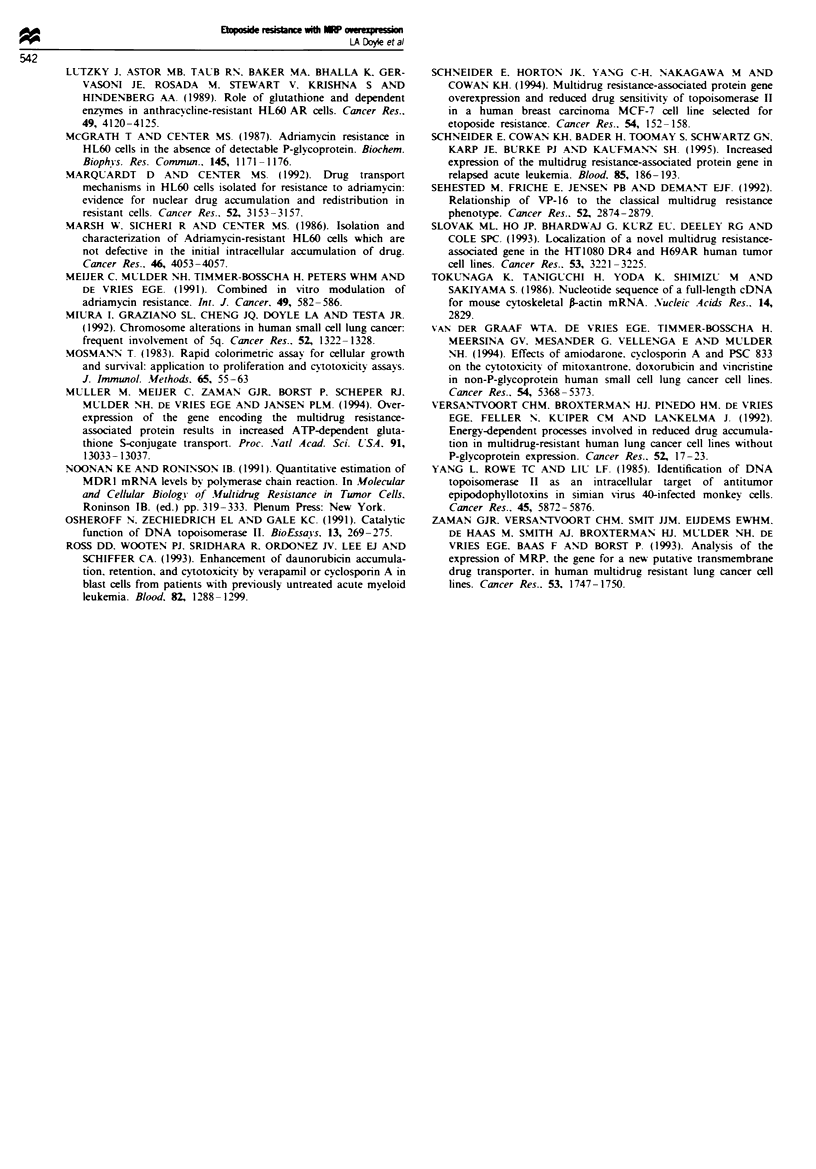# An etoposide-resistant lung cancer subline overexpresses the multidrug resistance-associated protein.

**DOI:** 10.1038/bjc.1995.370

**Published:** 1995-09

**Authors:** L. A. Doyle, D. D. Ross, J. V. Ordonez, W. Yang, Y. Gao, Y. Tong, C. P. Belani, J. C. Gutheil

**Affiliations:** Department of Medicine, University of Maryland School of Medicine, Baltimore, USA.

## Abstract

**Images:**


British Journal of Cancer (1995) 72, 535-542

© 1995 Stockton Press All rights reserved 0007-0920/95 $12.00

### An etoposide-resistant lung cancer subline overexpresses the multidrug resistance-associated protein

LA Doyle, DD Ross, JV Ordonez, W Yang, Y Gao, Y Tong, CP Belani and JC Gutheil

*Department of Medicine, University of Maryland School of Medicine and the University of Maryland Cancer Center, Baltimore, Maryland 21201, USA.*

**Summary** We have characterised an etoposide-resistant subline of the small-cell lung cancer cell line, UMCC-1, derived at our centre. Subline UMCC-1 VP was developed by culturing the parent line in increasing concentrations of etoposide over 16 months. UMCC-1 VP is 20-fold resistant to etoposide by MTT assays, relative to the parent line, and is cross-resistant to doxorubicin, vincristine and actinomycin D, but not to taxol, cisplatin, melphalan, thiotepa or idarubicin. Topoisomerase II immunoblotting demonstrates a 50% reduction of the protein in the resistant subline. The UMCC-1 VP subline demonstrates a marked decrease in the accumulation of [$^3$H]etoposide relative to the parent line, as well as a modest reduction in the accumulation of daunorubicin. Reverse transcription-polymerase chain reaction assays demonstrate no detectable *mdr1* expression but marked expression of the multidrug resistance-associated protein (MRP) gene in the resistant subline. Northern blotting with an MRP cDNA probe confirms marked overexpression of the MRP gene only in the UMCC-1 VP subline. Western blotting with antisera against MRP peptide confirms a 195 kDa protein band in the UMCC-1 VP subline. Southern blotting experiments demonstrate a 10-fold amplification of the MRP gene in the resistant subline. Depletion of glutathione with buthionine sulphoximine sensitised UMCC-1 VP cells to daunorubicin and etoposide. Our studies indicate that MRP gene expression may be induced by etoposide and may lead to reduced accumulation of the drug.

**Keywords**: drug resistance; multidrug resistance; lung cancer; etoposide; doxorubicin

In most patients with small-cell lung cancer (SCLC) tumours will respond initially to combination chemotherapy followed by a recurrence of cancer which is refractory to multiple cytotoxic agents (Harper *et al*., 1982). Multidrug-resistant SCLC tumours, like other bronchial carcinomas, have generally not been found to have overexpression of P-glycoprotein, a transmembrane protein which in other human tumours has been demonstrated to produce resistance by acting as an energy-dependent drug export transporter (Goldstein *et al*., 1989; Lai *et al*., 1989). The pattern of anti-cancer drug resistance seen in SCLC cells *in vitro* is often different from that of P-glycoprotein-mediated multidrug resistance (Giaccone *et al*., 1992; Jensen *et al*., 1993). Furthermore, multidrug resistance in cultured lung cancer cells is poorly reversed by chemosensitisers that are effective in cells which overexpress P-glycoprotein (Cole *et al*., 1989). There has been considerable recent interest in discovering the mechanisms underlying non-P-glycoprotein-mediated multidrug resistance in human lung cancer cells (Bergh *et al*., 1990; Doyle, 1993).

The gene for a novel drug transporter, termed the multidrug resistance-associated protein (MRP), was recently found to be amplified and overexpressed in a doxorubicin-selected SCLC cell line which is multiply drug resistant but does not overexpress P-glycoprotein (Cole *et al*., 1992). Several other doxorubicin-selected cancer cell lines have subsequently been found to have overexpression of the MRP gene (Slovak *et al*., 1993; Barrand *et al*., 1994). A full-length cDNA of MRP has been cloned, and two independent transfection experiments have demonstrated that cells transfected with MRP expression vectors demonstrate resistance to doxorubicin, etoposide and vincristine proportional to the levels of the 190 kDa membrane protein expressed by the MRP gene (Grant *et al*., 1994*a*; Kruh *et al*., 1994). Overexpression of MRP has recently been found to result in increased ATP-dependent glutathione *S*-conjugate transport (Jedlitschky *et al*., 1994; Muller *et al*., 1994). Increased expression of MRP has been found in relapsed acute leukaemia blasts, suggesting that MRP may contribute to clinical drug resistance in human cancer (Schneider *et al*., 1995).

Etoposide is one of the most clinically important drugs in the frontline treatment of SCLC tumours (Cavalli *et al*., 1978). The activity of etoposide results from a specific interaction of the drug with the nuclear enzyme topoisomerase II (Yang *et al*., 1985). This enzyme has the ability to alter the topological state of DNA and has a critical role in DNA replication, chromosomal segregation and RNA transcription (Liu, 1989; Osheroff *et al*., 1991). Topoisomerase II binds covalently to DNA and cleaves both strands, producing an intermediate termed the 'cleavable complex'. Drugs such as etoposide stabilise the cleavable complexes, and the double-stranded DNA breaks lead to cell death by, as yet, poorly defined mechanisms (Kaufmann, 1989). Drug resistance to topoisomerase II-directed agents can result from any mechanism that decreases the number of stabilised cleaved complexes. Studies of lung cancer cell lines with selective resistance to topoisomerase II-directed agents have detected both qualitative and quantitative alterations of the enzyme. While epipodophyllotoxin derivatives are usually included among the drugs recognised in the classic multidrug resistance phenotype, efflux studies indicate that these drugs are relatively poor substrates for P-glycoprotein (Sehested *et al*., 1992).

As models to study etoposide resistance in SCLC, we have derived the UMCC-1 VP and NCI-H1514 VP sublines by stepwise selection of the parent lines in etoposide. The purpose of our study was to characterise the drug resistance phenotype of the resistant sublines by determining their cross-resistance pattern, to investigate alterations in the target enzyme topoisomerase II and to examine changes in cellular accumulation of etoposide as well as the expression of membrane proteins implicated in the efflux of cytotoxic drugs.

### Materials and methods

### *Materials*

RPMI-1640 medium and fetal bovine serum were obtained from Gibco (Grand Island, NY, USA). [α-$^{32}$P]dCTP was

Correspondence: LA Doyle, University of Maryland Cancer Center, 22 South Greene Street, Baltimore, Maryland 21201, USA
Received 22 September 1994; revised 20 April 1995; accepted 27 April 1995.

obtained from Amersham (Arlington Heights, IL, USA) and [$^3$H]etoposide (specific activity, 900 mCi mmol$^{-1}$) was obtained from Morovek Laboratories (Brea, CA, USA). Etoposide and taxol were obtained from Bristol-Myers-Squibb (Syracuse, NY, USA), doxorubicin and idarubicin from Adria Laboratories (Columbus, OH, USA), daunorubicin from Wyeth Laboratories (Philadelphia, PA, USA), thiotepa from Lederle Laboratories (Pearl River, NY, USA), and verapamil, buthionine sulphoximine (BSO), vincristine, cisplatin, melphalan and actinomycin D from Sigma (St Louis, MO, USA). Cyclosporin A was kindly supplied by Sandoz (Basel, Switzerland). Polyclonal antisera against topoisomerase II was a generous gift from Dr LF Liu (UMDNJ Robert Wood Johnson Medical School, Piscataway, NJ, USA).

*Cell culture*

The NCI-H1514 cell line was a generous gift from Dr A Gazdar (Simmons Cancer Center, Dallas, TX, USA) and was obtained from an extensive-stage SCLC patient before the initiation of chemotherapy (Gazdar *et al.*, 1990). The UMCC-1 cell line was established from the bone marrow of a 64-year-old male patient with extensive SCLC treated at the University of Maryland Cancer Center. The marrow was obtained after the patient had relapsed following several different combinations of chemotherapy, including carboplatinum, etoposide, cyclophosphamide, doxorubicin, vinblastine, cisplatinum, methotrexate, vincristine and cyclohexylchloroethylnitrosourea (CCNU). The UMCC-1 cell line has a colony morphology and cytogenetic abnormalities characteristic of SCLC (Miura *et al.*, 1992)

The cell lines were maintained in RPMI-1640 medium containing 2 mM L-glutamine and 10% fetal bovine serum (Hyclone, Logan, UT, USA). Etoposide-resistant sublines of NCI-H1514 and UMCC-1 were selected by stepwise continuous exposure to increasing concentrations of etoposide, starting at 0.05 μM. Once the cells were adapted to a given concentration of etoposide the cultures were typically changed to a 2-fold increased concentration of the drug. Cells were not exposed to mutagens before selection. Each subline was subcloned by limiting dilution four times during a 16 month period. The resistant subline of NCI-H1514 is termed NCI-H1514 VP, and this subline was maintained continuously in 2 μM etoposide. The resistant subline of UMCC-1 is termed UMCC-1 VP. The UMCC-1 VP subline can survive continuous exposure to 16 μM etoposide for several weeks, but was chronically maintained at 4 μM etoposide. Both resistant sublines were maintained in etoposide until 7–14 days before the initiation of the individual experiments described. The leukaemia cell line HL-60, its doxorubicin-resistant subline HL-60 Adr and its vincristine-resistant subline HL-60 Vinc were generous gifts from Dr M Center of Kansas State University (Marsh *et al.*, 1986; McGrath and Center, 1987). The doxorubicin-resistant HL-60 AR subline was a generous gift from Dr A Hindenberg at the Winthrop University School of Medicine (Bhalla *et al.*, 1985).

*Chemosensitivity testing*

MTT (3-(4,5-dimethylthiazol-2-yl)-2,5-dipenyltetrazolium bromide) assays were performed with minor modifications of the method of Mosmann (1983). Briefly, 10 000 cells of each line were plated in 100 μl of medium in wells of 96-well microtitre plates for 24 h before the addition of drug. After overnight incubation at 37°C, appropriate concentrations of drug were added to each well. Drugs were made up in medium at five times the desired final concentration, and 25 μl of medium containing the drug stocks was added to each well. After a 72 h incubation at 37°C, 10 μl of MTT (5 mg ml$^{-1}$ stock) was added to each well. Plates were then incubated for an additional 4 h at 37°C, after which the medium was removed and the remaining formazan crystals solubilised in 100 μl of dimethylsulphoxide (DMSO). Plates were read on a Dynatek model 740 plate reader with a reference wavelength of 410 nm and a test wavelength of 490 nm. These data were downloaded into SigmaPlot (Jandel Scientific, San Rafael, CA, USA) for evaluation and graphing. The fold resistance is expressed as a ratio of the $IC_{50}$ of the resistant cell line relative to that of the sensitive cell line. Each drug was tested in at least two independent experiments, and within each experiment determinations were done in quadruplicate.

In sensitisation experiments, cells were incubated with 2–5 μM cyclosporin A or 10 μM verapamil for 30 min before the addition of etoposide. Cellular depletion of glutathione, in other experiments, was performed by the addition of 25–50 μM BSO to cells 24 h before the addition of daunorubicin or etoposide.

*Etoposide accumulation*

Etoposide accumulation was quantitated as previously described (Hamza *et al.*, 1987). A total of 4 × 10$^5$ cells was plated into T-25 flasks. After incubation for 2 days at 37°C the cells were harvested, washed and resuspended in RPMI-1640 medium with 10% fetal bovine serum (FBS), containing 0.5 μCi ml$^{-1}$ [$^3$H]etoposide (900 mCi mmol$^{-1}$) plus 10 μM unlabelled etoposide. Duplicate flasks were incubated for various times from 0 to 90 min then placed on ice. The cells were washed three times with cold phosphate-buffered saline (PBS) and the cell pellet was lyophilised overnight. The dried pellet was dissolved in 0.25 ml of 1 N sodium hydroxide for 4 h at room temperature, then neutralised with 0.25 ml of 1 N hydrochloric acid. One-third of the reaction mixture was used to determine protein concentration by the method of Lowry *et al.* (1951), with albumin as a standard, and the rest was processed for radioactivity with scintillation counting.

*Daunorubicin accumulation and retention*

Approximately 1 × 10$^6$ cells of each line were plated into T-25 flasks and incubated overnight in complete medium. The cells were exposed to 1 μg ml$^{-1}$ daunorubicin, and at the indicated time points intracellular daunorubicin accumulation was determined as previously described (Ross *et al.*, 1993). Daunorubicin fluorescence assays were performed on a flow cytometer (FACStar Plus, Becton Dickinson, San Jose, CA, USA) using laser excitation of 488 nm and reading fluorescence emission with the use of a 575-25 filter. Logarithmic amplification of red fluorescence signals was used throughout. Fluorescent beads (Propidium Iodide Alignment Microbead Standards, Flow Cytometry Standards Corporation, Research Triangle, NC, USA) were used to ensure reproducibility of the fluorescence measurements. Relative intracellular daunorubicin content for a particular sample was obtained by dividing the channel number that represented mean red fluorescence for that sample by 256 (the number of channels per log decade), then obtaining the antilog of this value. Ten thousand cells were used for each determination. Retention experiments were performed after 3 h of daunorubicin exposure by washing the cells three times in ice-cold PBS then determining the intracellular daunorubicin remaining after the indicated times of incubation. Daunorubicin fluorescence was plotted after subtracting background counts (0 time at 4°C) from all measurements.

*Western blot analysis*

Cellular lysates for topoisomerase II immunoblots were prepared as previously described (Kaufmann *et al.*, 1987). Cellular proteins were separated on 5–15% gradient sodium dodecyl sulphate (SDS)-polyacrylamide gels and transferred to nitrocellulose by electroblotting. The membranes were blocked in TSM buffer (0.15 M sodium chloride, 10 mM Tris–HCl pH 7.4, 5% skimmed milk). The blots were hybridised with a 1:500 dilution of rabbit antiserum against gel-purified topoisomerase II in TSM buffer overnight at room temperature, washed three times for 15 min each in TS buffer (0.15 M sodium chloride, 10 mM Tris–HCl pH 7.4) with 0.05% NP-40, then incubated for 90 min at room temperature with $^{125}$I-labelled goat anti-rabbit IgG (Amersham) in TSM buffer. The membranes were washed five times as before then developed for 6–24 h.

Western blotting for MRP was performed using detergent-solubilised membrane proteins as previously described (Chen *et al.*, 1990). The antiserum used was termed anti-MRP.6KQ by Dr N Krishnamachary, Kansas State University, and was generated against a peptide corresponding to MRP amino acids 246–260. The peptide had been conjugated to keyhole limpet haemocyanin, and the conjugate used to immunise New Zealand White rabbits (Krishnamachary and Center, 1993). Electrophoresis was performed using 50 μg of membrane protein per lane, and immunoblotting was performed with a 1:100 dilution of the antipeptide antisera. The blots were developed using a chemiluminescent detection system (ECL Kit, Amersham).

*Reverse transcription PCR assays*

One microgram of total RNA from each cell line, isolated using an RNA-STAT-60 kit (Tel-Test 'B', Friendswood, TX, USA), was reverse transcribed in 20 μl of RT buffer (10 mM Tris–HCl pH 8.0, 50 mM potassium chloride, 0.1% Triton X-100, 5 mM magnesium chloride, 250 μM each of dATP, dGTP, dCTP, dTTP), containing 20 units of RNasin and 10 units of AML reverse transcriptase (both from Promega), 500 ng of specific primers and 2 μg of yeast tRNA. Following incubation at 42°C for 45 min, the mixture was heated to 99°C for 5 min, then cooled to 4°C. The resulting cDNA mixture was serially diluted in RT buffer. In each dilution, the target sequences were amplified for 30 cycles by the polymerase chain reaction with specific primers for each gene, using 1.25 units of *Taq* DNA polymerase. The primers used were : *mdr1*, 5′-primer nt 2322–2346, 3′-primer nt 2512–2536; MRP, 5′-primer nt 3898–3914, 3′-primer nt 4454–4471; $\beta_2$-microglobulin, 5′-primer nt 1552–1571, 3′-primer nt 2252–2262 and nt 3207–3215 (Noonan and Roninson, 1991). Ten microlitres of the reaction products was separated on a 2% agarose gel in Tris–borate–EDTA buffer. The PCR reaction product bands were visualised by ethidium bromide staining.

*Northern blot analysis*

A 1 kb fragment from the 3′ region of MRP cDNA cloned into an *Eco*RI site in pGEM-3Zf(+), was obtained from Dr S Cole. RNA was prepared from cells using the RNA-STAT-60 kit. The cDNA clone, which was termed pmrp 10.1, was used for labelling by random priming with [α-$^{32}$P]dCTP (Prime-a-Gene labelling system, Promega) and probing of Northern blots. Twenty micrograms of total RNA was separated on a 1.2% agarose gel in formaldehyde/4-morpholinepropanesulphonic acid buffer. After transfer onto Immobilon-N polyvinylidene difluoride membranes (Millipore, Bedford, MA, USA), the membrane was prehybridised overnight and hybridised at 42°C for 20 h with the labelled MRP probe in 50% formamide. After washing, the membrane was exposed to X-ray film for 4–16 h at −70°C with intensifying screens. To estimate variation in RNA loading of the gel, the blots were stripped and rehybridised with a $^{32}$P-labelled cDNA probe from the 3′ region of mouse β-actin (Tokunaga *et al.*, 1986). Relative levels of MRP and β-actin mRNAs were determined by densitometry.

*Southern blot analysis*

Isolation of genomic DNA, agarose gel electrophoresis and DNA blot analysis followed standard procedures. Ten micrograms of genomic DNA from each line was digested with *Eco*RI and *Bam*HI and electrophoresed through a 0.8% agarose gel. After blotting onto a nitrocellulose membrane, prehybridisation was carried out for 4 h at 42°C in 50% formamide, 5 × standard saline phosphate–EDTA (1 × = 3 M sodium chloride, 0.2 M sodium dihydrogen phosphate, 0.02 M EDTA, pH 7.4), 0.5% SDS, 4 × Denhardt's and herring testes DNA (100 μg ml$^{-1}$). The blot was then hybridised for 20 h at 42°C with the pmrp 10.1 probe and labelled by random priming with [α-$^{32}$P]dCTP as described above. The blot was exposed to film and densitometry was carried out as before. The $^{32}$P-labelled MRP bands were also quantified on a Betascope 603 blot analyser (Betagen, Waltham, MA, USA).

### Results

*Cell line characteristics*

The UMCC-1/VP subline had a doubling time of 48 h and grew as loosely aggregated floating spheroids. The parental UMCC-1 line had a similar doubling time, but grew with a mixture of floating colonies and surface-adherent cells. The NCI-H1514 and NCI-H1514/VP sublines each had doubling times of approximately 60 h. The drug-resistant NCI-H1514/VP subline grew in less tightly compacted spheroid colonies than did the parental line.

*Drug resistance pattern*

The NCI-H1514 and UMCC-1 parental cell lines had comparable sensitivity to antineoplastic drugs, although they were derived from untreated and relapsed SCLC patients respectively (Tables I and II). The UMCC-1/VP subline was approximately 20-fold resistant to etoposide, relative to the UMCC-1 parent line, but also more than 10-fold resistant to doxorubicin, vincristine and actinomycin D (Table I). The NCI-H1514/VP subline was approximately 10-fold resistant to etoposide, relative to the parent line, but only 3-fold resistant to doxorubicin and not detectably resistant to actinomycin D or vincristine (Table II). The UMCC-1/VP cell line was also found to have no cross-resistance to taxol or thiotepa, and only 2-fold resistance to idarubicin, melphalan and cisplatin, relative to the UMCC-1 line (Table I).

*Drug accumulation and efflux*

The UMCC-1/VP subline had markedly reduced accumulation of etoposide compared with the sensitive parental line

**Table I** Sensitivity of UMCC-1 and UMCC-1/VP to cytotoxic drugs

| *Drug* | *UMCC-1* $IC_{50}$ (μM) ± s.e. | *UMCC-1/VP* $IC_{50}$ (μM) ± s.e. | *Fold* |
|---|---|---|---|
| Etoposide | 0.6153 ± 0.085 | 13.822 ± 1.83 | 22.5 |
| Doxorubicin | 0.0194 ± 0.0022 | 0.2452 ± 0.02 | 12.9 |
| Vincristine | 0.00074 ± 0.000065 | 0.0081 ± 0.0012 | 10.9 |
| Actinomycin D | 0.9349 ± 0.046 | 9.5252 ± 1.04 | 10.2 |
| Taxol | 0.0097 ± 0.0021 | 0.0073 ± 0.0016 | 0.78 |
| Melphalan | 14.1 ± 2.4 | 33.5 ± 6.9 | 2.4 |
| Idarubicin | 0.11 ± 0.038 | 0.22 ± 0.08 | 2.0 |
| Cisplatin | 2.5 ± 0.12 | 4.9 ± 0.017 | 1.95 |
| Thiotepa | 79.1 ± 17.3 | 82.6 ± 28.4 | 1.04 |

$IC_{50}$ concentrations were determined by the MTT assay as described in Materials and methods. Values represent the means ± s.e. of quadruplicate determinations in a representative experiment.

**Table II** Sensitivity of NCI-H1514 and NCI-H1514/VP to cytotoxic drugs

| *Drug* | *NCI-H1514* $IC_{50}$ (μM) ± s.e. | *NCI-H1514/VP* $IC_{50}$ (μM) ± s.e. | *Fold* |
|---|---|---|---|
| Etoposide | 0.7326 ± 0.091 | 9.8828 ± 1.52 | 13.5 |
| Doxorubicin | 0.0382 ± 0.0048 | 0.1273 ± 0.012 | 3.3 |
| Vincristine | 0.0012 ± 0.00015 | 0.0021 ± 0.00022 | 1.75 |
| Actinomycin D | 1.2001 ± 0.10 | 1.2839 ± 0.10 | 1.06 |

$IC_{50}$ concentrations were determined by the MTT assay as described in Materials and methods. Values represent the means ± s.e. of quadruplicate determinations in a representative experiment.

(Figure 1). The NCI-H1514/VP subline, in contrast, had levels of intracellular etoposide similar to the parental cells (data not shown). The UMCC-1/VP subline had a small but reproducible reduction in daunorubicin accumulation relative to the parental cells and had decreased retention of intracellular anthracycline, although the initial slopes of drug efflux from UMCC-1 and UMCC-1/VP were similar (Figure 2). The decreased retention of daunorubicin in the UMCC-1/VP subline relative to UMCC-1 was particularly marked after overnight incubation of the cells in drug-free medium.

*Topoisomerase II expression*

The resistance of NCI-H1514/VP to doxorubicin and etoposide but not vincristine or actinomycin D suggested an alteration in DNA topoisomerase II activity in this resistant subline. Immunoblot analysis with antisera against topoisomerase II, however, demonstrated only a modest difference in topoisomerase II protein levels between each of the two resistant sublines and their corrresponding drug-sensitive parental lines (Figure 3). Densitometric analysis revealed that UMCC-1/VP and NCI-H1514/VP each had an approximate 50% reduction in immunoreactive topoisomerase II content relative to the parental line. Preliminary studies of topoisomerase II activity from nuclear extracts of NCI-H1514/VP and UMCC-1/VP do not reveal significant differences in either relaxation of supercoiled DNA or etoposide-mediated DNA cleavage compared with the parental lines (data not shown).

*P-glycoprotein and MRP expression*

The relative resistance of UMCC-1/VP to vincristine and actinomycin D, as well as to doxorubicin and etoposide, suggested that cells of the resistant subline might overexpress *mdr1*. However, reverse transcription PCR assays demonstrated that neither UMCC-1/VP nor NCI-H1514/VP overexpressed *mdr1* mRNA (Figure 4). The predicted 190 bp band was seen in the HL-60/Vinc subline, which overexpresses *mdr1*, and with an *mdr1* cRNA control. A control $\beta_2$-microglobulin 120 bp band was demonstrable in each lane of a parallel PCR assay, indicating that the lack of *mdr1* expression in the lung cancer cells was not due to degraded RNA (Figure 4).

Reverse transcription PCR assays using MRP primers demonstrated that the UMCC-1/VP subline strongly expressed a predicted 575 bp MRP band to a level similar to that of the control HL-60/ADR subline, which is known to have amplification and overexpression of the MRP gene (Figure 5) (Krishnamachary and Center, 1993). Under the conditions used, no MRP expression was detectable in the parental UMCC-1 lane or in the NCI-H1514 and NCI-H1514/VP lanes, although control $\beta_2$-microglobulin bands

**Figure 1** Etoposide accumulation in UMCC-1 (○) and UMCC-1/VP (●) cells. Exponentially growing cells were incubated with 0.5 $\mu$Ci ml$^{-1}$ (900 mCi mmol$^{-1}$) [$^3$H]etoposide plus 10 $\mu$M unlabelled etoposide at 37°C. At the indicated time points the cells were washed, and radioactivity was determined by liquid scintillation counting. The results shown are the means ± s.e. from two separate experiments.

**Figure 2** Daunorubicin accumulation and retention in UMCC-1 (●) and UMCC-1/VP (○) cells. Cells were exposed to daunorubicin (1 $\mu$g ml$^{-1}$) for various times and daunorubicin intracellular content was determined by direct fluorescence of the drug by flow cytometry. Retention of daunorubicin after a 3 h accumulation was determined by washing the cells free of extracellular drug and culturing in drug-free medium. The remaining daunorubicin fluorescence was measured by flow cytometry at the times indicated.

**Figure 3** Western blotting for DNA topoisomerase II with extracts from leukaemia and lung cancer cells. The cellular lysates were separated on a 5–15% gradient SDS/polyacrylamide gel and transferred onto a nitrocellulose membrane. The membrane was incubated with a polyclonal rabbit antisera against topoisomerase II, followed by $^{125}$I-labelled goat anti-rabbit IgG. Lane 1, HL-60; lane 2, NCI-H1514; lane 3, NCI-H1514/VP; lane 4, UMCC-1; lane 5, UMCC-1/VP.

**Figure 4** Quantitative PCR assay for *mdr*1 and $\beta_2$-microglobulin. Total RNA was isolated from each cell line and reverse transcribed. Each resulting cDNA was amplified with primer pairs for *mdr*1 or $\beta_2$-microglobulin. Ten microlitres of each amplified reaction mixture was separated on a 2% agarose gel in Tris–borate–EDTA. The bands were visualised by ethidium bromide staining and photographed, with an expected 190 bp *mdr*1 PCR product and an expected 120 bp $\beta_2$-microglobulin PCR product. Lane 1, marker DNA; lane 2, no DNA added; lane 3, HL-60; lane 4, HL-60/Vinc; lane 5, *mdr*1 cRNA; lane 6, NCI-H1514; lane 7, NCI-H1514/VP; lane 8, UMCC-1; lane 9, UMCC-1/VP; lane 10, HL-60; lane 11, HL-60/Vinc; lane 12, NCI-H1514; lane 13, NCI-H1514/VP; lane 14, UMCC-1; lane 15, UMCC-1/VP.

were readily detectable in a PCR reaction using RNA from these lines (Figure 5).

The 575 bp PCR product was subsequently cut out of the gel and purified by phenol-chloroform extraction and ethanol precipitation. The DNA was sequenced on an Applied Biosystems automated sequencer, using the original PCR primer sequences as templates. DNA sequence analysis revealed that the PCR product sequence was consistent with authentic MRP (data not shown).

Relative overexpression of MRP in the UMCC-1/VP subline, suggested by reverse transcription PCR assays, was confirmed by Northern blotting experiments with an MRP cDNA probe (Figure 6). A marked increase in the 6.7 kb MRP RNA band, relative to the parental UMCC-1 line, was indicated by radioautography after a 4 h film exposure. No relative overexpression of MRP in the NCI-H1514/VP subline was demonstrated by Northern blotting.

Immunoblotting of the UMCC-1 and UMCC-1/VP sublines with antisera against an MRP peptide conjugate revealed the expected 195 kDa band in the resistant subline (Figure 7). Only a faint 195 kDa band was noted in the lane containing membrane proteins from the parental UMCC-1 line.

Amplification of the MRP gene in the UMCC-1/VP subline was demonstrable by Southern analysis (Figure 8). Betascope quantitation of $^{32}P$ emissions from bands on the MRP Southern blot demonstrated an 11-fold increase in counts from the UMCC-1 VP subline relative to the parental UMCC-1 line.

**Figure 5** Quantitative PCR assay for MRP in leukaemia and lung cancer cells. Total RNA was isolated from each cell line and reverse transcribed. Each resulting cDNA was amplified with primer pairs for MRP or $\beta_2$-microglobulin. Ten microlitres of each amplified reaction mixture was separated on a 2% agarose gel in Tris-borate-EDTA. The 575 bp MRP bands and 120 bp $\beta_2$-microglobulin bands were visualised by ethidium bromide staining and photographed. Lane 1, HL-60; lane 2, HL-60/ADR; lane 3, UMCC-1/VP; lane 4, UMCC-1; lane 5, NCI-H1514/VP; lane 6, NCI-H1514.

**Figure 6** Northern blotting for MRP expression in SCLC cell lines. Total cellular RNA (20 μg) from each cell line was separated on a 1.2% agarose gel, transferred to a PVDF membrane and hybridised with the radiolabelled pmrp 10.1 probe. The blot was later stripped and rehybridised with a control β-actin probe. Lane 1, UMCC-1/VP; lane 2, UMCC-1; lane 3, NCI-H1514/VP; lane 4, NCI-H1514.

**Figure 7** Western blotting for MRP expression in SCLC cell sublines. Fifty micrograms of detergent-solubilised membrane proteins from each line was separated by electrophoresis on 8-14% polyacrylamide gels and transferred to nitrocellulose. The blots were incubated with a 1:100 dilution of rabbit anti-MRP peptide antisera, and developed with a chemiluminescent detection system. Lane 1, UMCC-1; lane 2, UMCC-1 VP.

**Figure 8** Southern blot analysis of SCLC cell lines with an MRP probe. Ten micrograms of DNA from each line was digested with *Eco*RI and *Bam*HI, electrophoresed on a 0.8% agarose gel, transferred to a nitrocellulose filter and hybridised with the labelled pmrp 10.1 probe. Lane 1, NCI-H1514; lane 2, NCI-H1514 VP; lane 3, UMCC-1; lane 4, UMCC-1 VP.

*Modulation of drug resistance in UMCC-1 VP cells*

Verapamil, at a 10 μM concentration, produced a 4-fold sensitisation of UMCC-1 VP cells to etoposide in MTT assays (Table III). Cyclosporin A, at a 2 μM concentration, caused a 2-fold sensitisation to etoposide in UMCC-1 VP cells (Table III). Exposure of UMCC-1 VP cells to a 5 μM concentration of cyclosporin A for 72 h in the MTT assays resulted in unacceptable toxicity by the modulator (data not shown). Preincubation of UMCC-1 VP cells for 24 h by either 25 or 50 μM BSO caused a 2.4-fold sensitisation of the cells to etoposide and a 6.3-fold sensitisation to daunorubicin (Table III).

### Discussion

We have demonstrated MRP gene amplification and overexpression in a human SCLC subline made resistant *in vitro* to etoposide. The resistant subline UMCC-1/VP demonstrates resistance to other cytotoxic agents and decreased etoposide accumulation with no detectable expression of *mdr1*. The unaltered growth rate of the UMCC-1/VP cells, along with their vincristine resistance and the initial topoisomerase II characterisation, suggests that MRP overexpression may be the dominant mechanism of MDR in this subline. There is no explanation yet for the 10-fold resistance to etoposide demonstrated by the other subline NCI-H1514/VP. This subline has undetectable *mdr1* or MRP expression and unaltered etoposide levels. While the immunoreactive topoisomerase II protein was only reduced by 50% in NCI-H1514/VP, relative to the parent line, the pattern of drug resistance is consistent with an altered topoisomerase. Further topoisomerase II functional assays and sequence analysis for mutations are currently being conducted in the NCI-H1514/VP subline.

MRP amplification and overexpression has been noted in doxorubicin-resistant lung cancer cell lines such as the SCLC line GLC4/Adr and the non-small-cell lung cancer lines COR-L23/R and MOR/0.4R (Barrand *et al*., 1993, 1994; Zaman *et al*., 1993). MRP overexpression has also been demonstrated in anthracycline-selected leukaemia and fibrosarcoma sublines (Krishnamachary and Center, 1993; Slovak *et al*., 1993).

Antisera derived against synthetic MRP proteins demonstrate that the MRP protein is 195 kDa on immunoblots and enriched in membrane fractions (Krishnamachary and Center, 1993). Treatment of HL60/ADR cells wtih tunicamycin results in the appearance of a 165 kDa band reactive with the antipeptide serum (Krishnamachary and Center, 1993). We have used antipeptide antisera, obtained from Dr M Center, to demonstrate MRP protein in UMCC-1/VP membrane proteins by immunoblotting of one-dimensional and two-dimensional polyacrylamide gels and to reproduce the tunicamycin results in our cell line (manuscript in preparation).

The role of MRP in cellular drug accumulation is still undefined. Cole *et al*. (1992) originally reported little change in doxorubicin accumulation in the H69AR subline relative to the parental cells, but Coley *et al*. (1991) have found decreased levels of daunorubicin and vincristine in the MRP-overexpressing line COR-L23/R, relative to its parent line, after an initial delay of 30–60 min. The MRP-overexpressing cell lines GLC4/Adr and HL-60/Adr have also been found to have decreased anthracycline accumulation relative to the parental drug-sensitive cell lines (Marquardt and Center, 1992; Versantvoort *et al*., 1992). These results parallel our findings with the UMCC-1/VP subline, in which decreased daunorubicin accumulation and retention is most evident at later time points, suggesting a relatively slower efflux process. Most recently, MRP transfectants of HeLa cells have been demonstrated to have a modest decrease in vincristine accumulation (Grant *et al*., 1994*b*).

The UMCC-1/VP subline appears to demonstrate a greater relative decrease in etoposide accumulation than in the accumulation or retention of daunorubicin. Since the cells were selected in etoposide, mechanisms other than MRP may effect the transport of etoposide into this subline. Alternatively, since there is less non-specific membrane binding and DNA intercalation of epipodophyllotoxins than anthracyclines, the contribution of MRP to intracellular drug accumulation may be better seen with the former agents (Liu, 1989). Studies of etoposide accumulation in MRP-transfected cells will be useful in determining the role of MRP in the transport of this drug. Further experiments, using lower concentrations of anthracyclines, might also demonstrate greater differences in drug accumulation in MRP-overexpressing cells.

We have demonstrated a 4-fold sensitisation of UMCC-1/VP cells to etoposide by concomitant incubation with 10 μM verapamil. These findings are similar to the 4-fold sensitisation to vincristine and 9-fold sensitisation to daunorubicin by verapamil reported in L23/R cells, which overexpress MRP (Barrand *et al*., 1993). We could not demonstrate sensitisation of UMCC-1/VP cells to etoposide by non-toxic concentrations of cyclosporin A. Cyclosporin A has been previously demonstrated to have little effect on sensitisation to daunorubicin or doxorubicin in the MRP-overexpressing cell lines GLC4-Adr and HL60/AR (Gollapudi *et al*., 1992; van der Graaf *et al*., 1994).

We have demonstrated partial reversal of etoposide and daunorubicin resistance in UMCC-1/VP cells by pretreatment of the cells with BSO. MRP has been recently demonstrated to be an ATP-dependent glutathione *S*-conjugate transporter (Jedlitschky *et al*., 1994; Muller *et al*., 1994). The association between glutathione depletion by BSO and chemoresensitisation to daunorubicin or etoposide is unclear, because these drugs are not known to be conjugated to glutathione. However, BSO pretreatment has been demonstrated to increase anthracycline accumulation and cytotoxicity in several cell lines which overexpress MRP (Lutzky *et al*., 1989; Meijer *et al*., 1991; Gollapudi *et al*., 1992; Longhurst *et al*., 1994).

While most cancer cell lines overexpressing MRP have been selected with anthracyclines, MRP overexpression has been recently reported in an MCF-7 breast cancer subline

**Table III** Effects of cyclosporin A, verapamil and BSO on sensitivity of UMCC-1/VP cells to daunorubicin and etoposide

| | Modifier | | | |
|---|---|---|---|---|
| | *None* | *Cyclosporin A* | *Verapamil* | *BSO* |
| Etoposide | | | | |
| $IC_{50}$ | 28.3 ± 11.6 | 13.1 ± 2.4 | 7.3 ± 2.6 | 11.8 ± 2.0 |
| Sensitisation ratio | 1.0 | 2.2 | 3.9 | 2.4 |
| Daunorubicin | | | | |
| $IC_{50}$ | 0.300 ± 0.55 | NT | NT | 0.048 ± 0.0078 |
| Sensitisation ratio | 1.0 | | | 6.3 |

$IC_{50}$ values are shown as the mean ± s.e. calculated from data obtained in two experiments, each based on determinations from four wells. Sensitisation ratios were calculated as $IC_{50}$ without modifier/$IC_{50}$ with modifier. Cyclosporin A was 2 μM and verapamil was 10 μM. Cells were pretreated with 25 μM BSO for 24 h before adding etoposide or daunorubicin.

selected in etoposide (Schneider *et al*., 1994). This subline also demonstrated decreased accumulation of etoposide relative to the parent cells in the absence of detectable *mdr*1 expression. The expression of MRP by the UMCC-1/VP subline appears to be greater than that of the MCF-7/VP line reported, since Northern blotting experiments easily detected MRP in UMCC-1/VP after a 4 h film exposure, while a 14 day exposure was used for the MCF-7/VP subline. While these two sublines have approximately the same relative resistance to etoposide and doxorubicin, the UMCC-1/VP subline was more resistant to vincristine and had a greater apparent decrease in etoposide accumulation. The differences in MDR phenotype between these two etoposide-resistant sublines may be related to concomitant changes in topoisomerase II activity during the drug selection process. The UMCC-1/VP subline confirms that MRP gene amplification and overexpression can occur during etoposide selection of resistant cells, and suggests that MRP overexpression may contribute to the atypical multidrug resistance seen after etoposide-based induction chemotherapy in bronchial malignancies.

**Abbreviations**: MDR, multidrug resistance; P-gp, P-glycoprotein; MRP, multidrug resistance-associated protein; WT, wild type; PBS, phosphate-buffered saline; SDS, sodium dodecyl sulphate; $IC_{50}$, drug concentration that inhibits cell growth by 50% under the assay conditions used; SCLC, small-cell lung cancer; MTT, 3-(4,5-dimethylthiazol-2-yl)2,5-diphenyltetrazolium bromide; AML, acute myelogenous leukaemia; BSO, buthionine sulphoximine.

**Acknowledgements**
We would like to thank Drs SPC Cole and CE Grant for helpful suggestions and the gift of MRP cDNA, and Dr LF Liu for the gift of topoisomerase II antisera. We are indebted to Drs A Hindenberg, AF Gazdar and M Center for gifts of cell lines, and to Drs Center and N Krishnamachary for the gift of MRP antisera. We would also like to thank Ms F Wade and Ms H Spiker for help in preparing the manuscript. This study was supported by the Bristol-Myers-Squibb Corporation under a research grant programme for studies of tumour resistance to chemotherapy.

### References

BARRAND MA, RHODES T, CENTER MS AND TWENTYMAN PR. (1993). Chemosensitization and drug accumulation effects of cyclosporin A, PSC-833 and verapamil in human MDR large cell lung cancer cells expressing a 190 k membrane protein distinct from P-glycoprotein. *Eur. J. Cancer*, **29A**, 408-415.

BARRAND MA, HEPPELL-PARTON AC, WRIGHT KA, RABBITTS PH AND TWENTYMAN PR. (1994). A 190-kilodalton protein overexpressed in non-P-glycoprotein-containing multidrug-resistant cells and its relationship to the MRP gene. *J. Natl Cancer Inst.*, **86**, 110-117.

BERGH J, NYGREN P AND LARSSON R. (1990). Mechanisms for acquired cytotoxic drug resistance in human small cell lung cancer and the potential utilization of resistance modifiers - a review with focus on *in vitro* studies. *Lung Cancer*, **6**, 9-15.

BHALLA K, HINDENBERG A, TAUB RN AND GRANT S. (1985). Isolation and characterization of an anthracycline-resistant human leukemic cell line. *Cancer Res.*, **45**, 3657-3662.

CAVALLI F, SONNTAG R, JUNGI F, SENN HJ AND BRUNNER KW. (1978). VP-16-213 monotherapy for remission induction of small cell lung cancer: a randomized trial using three dosage schedules. *Cancer Treat. Rep.*, **62**, 473-475.

CHEN Y-N, MICKLEY LA, SCHWARTZ AM, ACTON EM, HWANG J AND FOJO AT. (1990). Characterization of adriamycin-resistant human breast cancer cells which display overexpression of a novel resistance-related membrane protein. *J. Biol. Chem.*, **265**, 10073-10080.

COLE SPC, DOWNES HF AND SLOVAK ML. (1989). Effect of calcium antagonists on the chemosensitivity of two multidrug resistant human tumour cell lines which do not overexpress P-glycoprotein. *Br. J. Cancer*, **59**, 42-46.

COLE SPC, BHARDWAJ G, GERLACH JH, MACKIE JE, GRANT CE, ALMQUIST KC, STEWART AJ, KURZ EU, DUNCAN AMV AND DEELEY RG. (1992). Overexpression of a transporter gene in a multidrug-resistant human lung cancer cell line. *Science*, **258**, 1650-1654.

COLEY HM, WORKMAN P AND TWENTYMAN PR. (1991). Retention of activity by selected anthracyclines in a multidrug resistant human large cell lung carcinoma line without P-glycoprotein hyperexpression. *Br. J. Cancer*, **63**, 351-357.

DOYLE LA. (1993). Mechanisms of drug resistance in human lung cancer cells. *Semin. Oncol.*, **20**, 326-337.

GAZDAR AF, STEINBERG SM, RUSSELL EK, LINNOILA RI, OIE HK, GHOSH BC, COTELINGEN JD, JOHNSON BE, MINNA JD AND IHDE DC. (1990). Correlation of in vitro drug-sensitivity testing results with response to chemotherapy and survival in extensive-stage small cell lung cancer: a prospective clinical trial. *J. Natl Cancer Inst.*, **82**, 117-124.

GIACCONE G, GAZDAR AF, BECK H, ZUNINO F AND CAPRANICO G. (1992). Multidrug sensitivity phenotype of human lung cancer cells associated with topoisomerase II expression. *Cancer Res.*, **52**, 1666-1674.

GOLDSTEIN LJ, GALSKI H, FOJO A, WILLINGHAM M, LAI SL, GAZDAR A, PIRKER R, GREEN A, CRIST W AND BRODEUR GM. (1989). Expression of a multidrug resistance gene in human cancers. *J. Natl Cancer Inst.*, **81**, 116-123.

GOLLAPUDI S AND GUPTA S. (1992). Lack of reversal of daunorubicin resistance in HL60/AR cells by cyclosporin A. *Anticancer Res.*, **12**, 2127-2132.

GRANT CE, VALDIMARSSON G, HIPFNER DR, ALMQUIST KC, COLE SPC AND DEELEY RG. (1994*a*). Overexpression of multidrug resistance-associated protein (MRP) increases resistance to natural product drugs. *Cancer Res.*, **54**, 357-361.

GRANT CE, VALDIMARSSON G, HIPFNER DR, ALMQUIST KC, COLE SPC AND DEELEY RG. (1994*b*). Overexpression of multidrug resistance-associated protein and resistance to natural product drugs (abstract). *Proc. Am. Assoc. Cancer Res.*, **35**, 54.

HAMZA M, CANAL P, BUGAT R, SOULA G AND DOUSTE-BLAZY L. (1987). Uptake and binding of teniposide (VM 26) in Krebs II ascites cells. *Biochem. Pharmacol.*, **36**, 1599-1603.

HARPER PG, DALLY MB AND GEDDES DM. (1982). Epipodophyllotoxin (VP-16) in small cell carcinoma of the bronchus resistant to initial combination chemotherapy. *Cancer Chemother. Pharmacol.*, **7**, 179-180.

JEDLITSCHKY G, LEIER I, BUCHHOLZ U, CENTER M AND KEPPLER D. (1994). ATP-dependent transport of glutathione S-conjugates by the multidrug resistance-associated protein. *Cancer Res.*, **54**, 4833-4836.

JENSEN PB, CHRISTENSEN IJ, SEHESTED M, HANSEN HH AND VINDELOV L. (1993). Differential cytotoxicity of 19 anti-cancer agents in wild-type and etoposide-resistant lung cancer cell lines. *Br. J. Cancer*, **67**, 311-320.

KAUFMANN SH. (1989). Induction of endonucleolytic DNA cleavage in human acute myelogenous leukemia cells by etoposide, camptothecin, and other cytotoxic anticancer drugs: a cautionary note. *Cancer Res.*, **49**, 5870-5878.

KAUFMANN SH, EWING CM AND SHAPER JH. (1987). The erasable Western blot. *Anal. Biochem.*, **161**, 89-95.

KRISHNAMACHARY N AND CENTER MS. (1993). The MRP gene associated with a non-P-glycoprotein multidrug resistance encodes a 190-kDa membrane bound glycoprotein. *Cancer Res.*, **53**, 3658-3661.

KRUH GD, CHAN A, MYERS K, GAUGHAN K, MIKI T AND AARONSON SA. (1994). Expression complementary DNA library transfer establishes mrp as a multidrug resistance gene. *Cancer Res.*, **54**, 1649-1652.

LAI S-L, GOLDSTEIN LJ, GOTTESMAN MM, PASTAN I, TSAI CM, JOHNSON BE, MULSHINE JL, IHDE DC, KAYSER K AND GAZDAR AF. (1989). MDR1 gene expression in lung cancer. *J. Natl Cancer Inst.*, **81**, 1144-1150.

LIU LF. (1989). DNA topoisomerase poisons as antitumor drugs. *Annu. Rev. Biochem.*, **58**, 351-375.

LONGHURST T, HARVIE RM, DAVEY MW AND DAVEY RA. (1994). Multidrug resistance mechanisms in epirubicin selected CCRF-CEM human leukemia cells which express mrp (abstract). *Proc. Amer. Assoc. Cancer Res.*, **35**, 75.

LOWRY OH, ROSEBROUGH NJ, FARR AL AND RANDALL RJ. (1951). Protein measurement the Folin phenol reagent. *J. Biol. Chem.*, **193**, 265-275.

LUTZKY J, ASTOR MB, TAUB RN, BAKER MA, BHALLA K, GERVASONI JE, ROSADA M, STEWART V, KRISHNA S AND HINDENBERG AA. (1989). Role of glutathione and dependent enzymes in anthracycline-resistant HL60 AR cells. *Cancer Res.*, **49**, 4120–4125.

McGRATH T AND CENTER MS. (1987). Adriamycin resistance in HL60 cells in the absence of detectable P-glycoprotein. *Biochem. Biophys. Res. Commun.*, **145**, 1171–1176.

MARQUARDT D AND CENTER MS. (1992). Drug transport mechanisms in HL60 cells isolated for resistance to adriamycin: evidence for nuclear drug accumulation and redistribution in resistant cells. *Cancer Res.*, **52**, 3153–3157.

MARSH W, SICHERI R AND CENTER MS. (1986). Isolation and characterization of Adriamycin-resistant HL60 cells which are not defective in the initial intracellular accumulation of drug. *Cancer Res.*, **46**, 4053–4057.

MEIJER C, MULDER NH, TIMMER-BOSSCHA H, PETERS WHM AND DE VRIES EGE. (1991). Combined in vitro modulation of adriamycin resistance. *Int. J. Cancer*, **49**, 582–586.

MIURA I, GRAZIANO SL, CHENG JQ, DOYLE LA AND TESTA JR. (1992). Chromosome alterations in human small cell lung cancer: frequent involvement of 5q. *Cancer Res.*, **52**, 1322–1328.

MOSMANN T. (1983). Rapid colorimetric assay for cellular growth and survival: application to proliferation and cytotoxicity assays. *J. Immunol. Methods*, **65**, 55–63

MULLER M, MEIJER C, ZAMAN GJR, BORST P, SCHEPER RJ, MULDER NH, DE VRIES EGE AND JANSEN PLM. (1994). Overexpression of the gene encoding the multidrug resistance-associated protein results in increased ATP-dependent glutathione S-conjugate transport. *Proc. Natl Acad. Sci. USA*, **91**, 13033–13037.

NOONAN KE AND RONINSON IB. (1991). Quantitative estimation of MDR1 mRNA levels by polymerase chain reaction. In *Molecular and Cellular Biology of Multidrug Resistance in Tumor Cells*, Roninson IB. (ed.) pp. 319–333. Plenum Press: New York.

OSHEROFF N, ZECHIEDRICH EL AND GALE KC. (1991). Catalytic function of DNA topoisomerase II. *BioEssays*, **13**, 269–275.

ROSS DD, WOOTEN PJ, SRIDHARA R, ORDONEZ JV, LEE EJ AND SCHIFFER CA. (1993). Enhancement of daunorubicin accumulation, retention, and cytotoxicity by verapamil or cyclosporin A in blast cells from patients with previously untreated acute myeloid leukemia. *Blood*, **82**, 1288–1299.

SCHNEIDER E, HORTON JK, YANG C-H, NAKAGAWA M AND COWAN KH. (1994). Multidrug resistance-associated protein gene overexpression and reduced drug sensitivity of topoisomerase II in a human breast carcinoma MCF-7 cell line selected for etoposide resistance. *Cancer Res.*, **54**, 152–158.

SCHNEIDER E, COWAN KH, BADER H, TOOMAY S, SCHWARTZ GN, KARP JE, BURKE PJ AND KAUFMANN SH. (1995). Increased expression of the multidrug resistance-associated protein gene in relapsed acute leukemia. *Blood*, **85**, 186–193.

SEHESTED M, FRICHE E, JENSEN PB AND DEMANT EJF. (1992). Relationship of VP-16 to the classical multidrug resistance phenotype. *Cancer Res.*, **52**, 2874–2879.

SLOVAK ML, HO JP, BHARDWAJ G, KURZ EU, DEELEY RG AND COLE SPC. (1993). Localization of a novel multidrug resistance-associated gene in the HT1080 DR4 and H69AR human tumor cell lines. *Cancer Res.*, **53**, 3221–3225.

TOKUNAGA K, TANIGUCHI H, YODA K, SHIMIZU M AND SAKIYAMA S. (1986). Nucleotide sequence of a full-length cDNA for mouse cytoskeletal β-actin mRNA. *Nucleic Acids Res.*, **14**, 2829.

VAN DER GRAAF WTA, DE VRIES EGE, TIMMER-BOSSCHA H, MEERSINA GV, MESANDER G, VELLENGA E AND MULDER NH. (1994). Effects of amiodarone, cyclosporin A and PSC 833 on the cytotoxicity of mitoxantrone, doxorubicin and vincristine in non-P-glycoprotein human small cell lung cancer cell lines. *Cancer Res.*, **54**, 5368–5373.

VERSANTVOORT CHM, BROXTERMAN HJ, PINEDO HM, DE VRIES EGE, FELLER N, KUIPER CM AND LANKELMA J. (1992). Energy-dependent processes involved in reduced drug accumulation in multidrug-resistant human lung cancer cell lines without P-glycoprotein expression. *Cancer Res.*, **52**, 17–23.

YANG L, ROWE TC AND LIU LF. (1985). Identification of DNA topoisomerase II as an intracellular target of antitumor epipodophyllotoxins in simian virus 40-infected monkey cells. *Cancer Res.*, **45**, 5872–5876.

ZAMAN GJR, VERSANTVOORT CHM, SMIT JJM, EIJDEMS EWHM, DE HAAS M, SMITH AJ, BROXTERMAN HJ, MULDER NH, DE VRIES EGE, BAAS F AND BORST P. (1993). Analysis of the expression of MRP, the gene for a new putative transmembrane drug transporter, in human multidrug resistant lung cancer cell lines. *Cancer Res.*, **53**, 1747–1750.